# Impacts of replanting American ginseng on fungal assembly and abundance in response to disease outbreaks

**DOI:** 10.1007/s00203-021-02196-8

**Published:** 2021-02-22

**Authors:** Li Ji, Lei Tian, Fahad Nasir, Jingjing Chang, Chunling Chang, Jianfeng Zhang, Xiujun Li, Chunjie Tian

**Affiliations:** 1 Key Laboratory of Mollisols Agroecology, Northeast Institute of Geography and Agroecology, Chinese Academy of Sciences, Changchun, 130102 Jilin China; 2 University of Chinese Academy of Sciences, Beijing, 100049 China; 3 Key Laboratory of Straw Biology and Utilization of the Ministry of Education, Jilin Agricultural University, Changchun, 130118 Jilin China

**Keywords:** Continuous cropping, Fungal community, Old ginseng field, Pathogens

## Abstract

Soil physicochemical properties and fungal communities are pivotal factors for continuous cropping of American ginseng (*Panax quinquefolium* L.). However, the response of soil physicochemical properties and fungal communities to replant disease of American ginseng has not yet been studied. High-throughput sequencing and soil physicochemical analyses were undertaken to investigate the difference of soil fungal communities and environmental driver factors in new and old ginseng fields; the extent of replant disease in old ginseng fields closely related to changes in soil properties and fungal communities was also determined. Results indicated that fungal communities in an old ginseng field were more sensitive to the soil environment than those in a new ginseng field, and fungal communities were mainly driven by soil organic matter (SOM), soil available phosphorus (AP), and available potassium (AK). Notably, healthy ginseng plants in new and old ginseng fields may influence fungal communities by actively recruiting potential disease suppressive fungal agents such as *Amphinema*, *Cladophialophora*, *Cadophora*, *Mortierella,* and *Wilcoxina*. When these key groups and members were depleted, suppressive agents in the soil possibly declined, increasing the abundance of pathogens. Soil used to grow American ginseng in the old ginseng field contained a variety of fungal pathogens, including *Alternaria*, *Armillaria*, *Aphanoascus*, *Aspergillus*, *Setophoma,* and *Rhexocercosporidium*. Additionally, micro-ecological factors affecting disease outbreaks in the old ginseng field included a strengthening in competition relationships, a weakening in cooperation relationships, and a change of trophic strategies among fungal communities.

**Supplementary Information:**

The online version contains supplementary material available at 10.1007/s00203-021-02196-8.

## Introduction

American ginseng (*Panax quinquefolium* L.) is a perennial herbaceous plant that has been used in herbal remedies around the world (Christensen et al. 2006; Dong et al. 2017). However, due to the relatively long time taken for American ginseng to mature (4 years) (Schmidt et al. 2019), the increase in demand for this herb and the lack of available fields has led to American ginseng crops to be replanted in the same location/field. Compared to newly cultivated fields, old ginseng fields (the field where ginseng plants had been previously cultivated and harvested) are characterized by problems associated with replanting diseases, resulting in lower yields and plants having poor medicinal quality (e.g., ginsenosides) (He et al. 2009; Rahman and Punja 2005; Wang 2017). Generally, after 3 years of growth in an old ginseng field, the survival rate of cultivated ginseng seedlings is less than 25%; about 75% of ginseng plants suffer from various diseases, including fibrous root fall-off and rotten roots (Wu et al. 2008). Given that the replant failure of American ginseng is closely related to disease outbreak, mechanisms underlying problems associated with replanted American ginseng urgently need to be identified to properly address disease issues related to replanting.

Previous studies have shown that both abiotic and biotic soil factors affect continuous cropping of American ginseng (Dong et al. 2017; Fu et al. 2009). For abiotic factors, replanting failure has been closely linked to changes in soil physical and chemical properties (Bennett et al. 2012; Li et al. 2020a, b). For example, Liu et al. (2020) demonstrated that continuous cropping of American ginseng significantly decreased rhizosphere soil pH, ammonium, available phosphorus, and available potassium contents. Since changes in soil properties are closely related to soil microbial communities (Lauber et al. 2008; Lei et al. 2020; Li et al. 2018), replanting failure is also related to soil biotic factors, including fungal communities. A recent study by Jiang et al. (2019) showed that continuous cropping of American ginseng could significantly decrease soil fungal richness and diversity, and increase the abundance of fungal pathogens (especially the genera *Monographella*) in root-rot diseased plants compared to healthy plants. Similarly, Dong et al. (2016) reported that continuous cropping of *Panax notoginseng* (same genus as American ginseng) could significantly decrease fungal diversity while increasing the relative abundance of *Fusarium* sp*.* pathogens, which was positively associated with *P. notoginseng* death rates. Additionally, the majority of soil-borne diseases in American ginseng are caused by a diverse array of fungal pathogens such as *Fusarium* spp. and *Cylindrocarpon* spp. (Rahman and Punja 2007). Although fungal pathogens have been linked to outbreaks of replanting diseases in many crops (Wang et al. 2018a, b; Yang et al. 2012), it is still unclear if fungal pathogens have a negative legacy, contributing to the outbreak of American ginseng replanting diseases in old ginseng fields.

In this study, we hypothesize that: (1) the legacy effect in an old ginseng field will lead to differences in soil physical and chemical properties between new and old ginseng fields; (2) the composition of fungal communities in new and old ginseng fields are driven by different soil properties; and (3) differences in response to disease are associated with diversity, interactions, and the function of fungal communities in new and old ginseng fields. Results gained from this study provide a basis to expand our understanding of the soil fungal environment and disease management of American ginseng in old ginseng fields.

## Materials and methods

### Sampling site description and sampling methods

The sampling site is located in Ji’an City, an experimental base for the re-use of old ginseng field (126°11′33″ E, 41°10′2″ N; 250 m altitude), Jilin Province, China. This area is characterized by a dark brown soil, an annual average temperature of 6.5 ℃, average annual precipitation between 800 and 1000 mm, and a frost-free period of about 150 days. The old ginseng field selected for study was cultivated with American ginseng for 4 years; the crop was harvested in the 5th year (in September 2015). In contrast, a field that had not previously been cultivated with American ginseng was selected as a new ginseng field. The distance between the new and old ginseng fields is about 200 m. In October 2016, American ginseng seeds were sown at the same time in the new and old ginseng fields and then covered with crushed corn straw and cold-proof film. The same agronomic management and fertilization regimes were applied for both fields, including the application of polyoxin spray and metalaxyl agrochemicals according to the manufacturers’ instructions, and 46.2 g/m^2^ potassium sulfate compound fertilizer and potassium dihydrogen phosphate foliar fertilizer.

Soil samples surrounding 3-year-old American ginseng plants in the new and old ginseng fields were collected (August 2019) by removing plants from a depth of 20 cm and gently shaking the soil from the roots. According to observations, about 2 weeks before sample collection some plants had symptoms of withered yellow leaves and rotten roots in both the new and old ginseng fields. Although the disease symptoms were almost the same in both (new and old) fields, the number of infected plants was remarkably higher in the old ginseng field than in the new ginseng field. Soil samples from plants with the same disease symptoms in both fields were labeled as ND and OD, respectively. Soil samples from plants with green stems and leaves without any disease symptoms in both fields were labeled as NH and OH, respectively. Blank control samples were obtained by collecting soil from a depth of 0–20 cm from uncultivated American ginseng soil in both the new and old ginseng fields, labeled as NB and OB, respectively. For each soil sample four replicas were analyzed, and for each replication five random soil samples were pooled together. Soil samples were sealed in airtight plastic bags and stored in dry-ice before being transported to the laboratory. All samples were homogenized and divided into two sub-samples: one sub-sample was air-dried at room temperature for soil property analysis, and the other was stored at − 80 °C for DNA extraction.

### Soil property analyses and DNA extraction

Soil pH (PHS-3C, Shang Hai Shengci Instrument Co., Ltd, China) and electrical conductivity (EC; DDS 11A, Shang Hai Yoke Instrument Co., Ltd, China) were measured using a soil:water ratio of 1:5 (dry weight/volume). Soil total phosphorus (TP), total nitrogen (TN), and soil organic matter (SOM) were measured following the methods of Luo et al. (2018). Soil available phosphorus (AP), available potassium (AK), and KMnO_4_-oxidizable carbon (EOC) were evaluated using the methods of Luo et al. (2017) and Shi et al. (2019). Total DNA was extracted from 0.5 g of soil using a Fast DNA SPIN Kit (MP Biomedicals, Santa Ana, CA, USA) according to the manufacturer’s instructions.

### ITS gene amplification and purification

DNA concentrations were measured using a NanoDrop 2000 spectrophotometer (NanoDrop Technologies, Inc., Wilmington, DE, USA), and extracted DNA was used as the template for the polymerase chain reaction (PCR). Internal transcribed spacer (ITS) amplicons were produced for each sample using barcoded primers. The primer pair used to amplify the fungal ITS region was ITS5F (5′-GGAAGTAAAAGTCGTAACAAGG-3′) and ITS1R (5′-GCTGCGTTCTTCATCGATGC-3′) (Chang et al. 2019). PCR was performed in a 25 μl mixture containing 5 × reaction buffer 5 µl, 5 × GC buffer 5 µl, dNTP (2.5 mM) 2 µl, forward primer (10 µM) 1 µl, reverse primer (10 µM) 1 µl, DNA template 2 µl, sterile double-distilled water 8.75 µl, and Q5 DNA polymerase 0.25 µl. The PCR products were purified using a Qiagen PCR Purification Kit (Qiagen, Inc., Shanghai, China) and pooled in equimolar concentrations.

### Illumina HiSeq sequence processing

High-throughput sequencing was performed using an Illumina MiSeq platform (Biomarker Technologies Co. Ltd., Beijing, China), following the methods of Xu et al. (2019). In summary, raw paired-end sequences were joined using FLASH (v1.2.7) (Magoč and Salzberg 2011), after which adapters and unique (i.e., singleton) sequences were removed by filtering raw tags using Trimmomatic (v0.33). Clean tags were obtained by undertaking quality filtering and dereplication (Bolger et al. 2014). Chimeric sequences were removed using UCHIME (v4.2), and effective tags were obtained (Edgar et al. 2011). UCLUST v1.2.22 was used to classify operational taxonomic units (OTUs) at the 97% similarity level (Edgar 2013). Taxonomy was assigned for each phylotype using the Ribosomal Database Project (RDP) classifier based on the UNITE database (http://unite.ut.ee/index.php) for fungi (Abarenkov et al. 2010).

### Statistical analyses

Based on our raw data, fungal diversity analysis was performed using BMKCloud (http://www.biocloud.net). In summary, differences in α-diversity indices (Shannon index, Simpson index, Chao1 index, ACE index) and soil properties were assessed via one-way analysis of variance (ANOVA; Tukey’s multiple comparison test) using IBM SPSS v20.0 statistical software (SPSS, Chicago, IL, USA). Fungal OTUs shared among compartments were analyzed using the “Venn Diagram” package in R (v3.4.0) (https://www.r-project.org). Community patterns were analyzed using Principal Coordinate Analysis (PCoA) based on Bray–Curtis distance in the “vegan” package (Sakaki et al. 1994). Relationships between soil properties and fungal communities were examined using redundancy analysis (RDA) in the “vegan” package (v2.4.1) in R v3.2.1 (McMurdie and Holmes 2013).

Differences in relative abundance at phylum and class levels were calculated using an ANOVA test. All data having differences at *P* < 0.05 were considered statistically significant. Biomarkers within different groups were quantitatively analyzed using linear discriminant analysis (LDA) effect size (LEfSe) analysis based on a normalized relative abundance matrix (Segata et al. 2011). LEfSe uses the Kruskal–Wallis rank sum test to detect features with significantly different abundances of assigned taxa and performs LDA to estimate the effect size of each feature.

A co-occurrence network was constructed using pairwise Spearman’s rank correlation coefficients at the genus level (Spearman’s rho > 0.9, *P* < 0.05). Network topology characteristics were calculated using Gephi, as well as visualizing the network data (Bastian et al. 2009). Ecological guilds of fungal communities were assigned using FUNGuild v1.0 (Fungi Functional Guild) (http://funguild.org) (Nguyen et al. 2016).

### Data accessibility

Fungal raw sequence data, deposited in the National Center for Biotechnology Information (NCBI), can be accessed using the accession number: PRJNA634884.

## Results

After quality filtering of the sequences, a total of 623,150 effective tags were clustered into 870 OTUs at the 97% sequence identity in the fungal community. The number of effective tags per sample ranged from 76,410 to 127,313, and the average length ranged from 247 to 258 bp (Tables S1, S2). As rarefaction curves approached the saturation plateau, sequencing coverage was sufficient to detect the majority of species (Fig. S1).

### Soil physicochemical properties

Differences in soil physicochemical properties between new and old ginseng fields were clarified by observing changes in soil properties (Table 1). Here, pH and TN of ginseng-grown soil samples in the old ginseng field were significantly lower than those in the new ginseng field; AK of the ginseng-grown soil samples in the old ginseng field was significantly higher than that in the new ginseng field. Moreover, soil pH, EC, and AK were significantly higher in ND samples than those in NH samples. SOM in OD was significantly lower compared to OH, and soil AK content was significantly higher in OD with respect to other samples.

**Table 1:** Differences in soil properties between new and old ginseng fields

	*N*			*O*		
	NB	ND	NH	OB	OD	OH
pH	6.62 ± 0.03a	6.71 ± 0.06a	6.4 ± 0.06b	5.82 ± 0.08c	5.77 ± 0.01c	5.94 ± 0.09c
EC (µs/cm)	85.7 ± 1.71a	82.93 ± 2.83a	55.27 ± 6.76b	31.07 ± 1.96c	58.53 ± 2.38b	62.43 ± 2.45b
SOM (g/kg)	28.49 ± 1.63ab	29.58 ± 1.66ab	30.99 ± 1.35a	26.93 ± 1.24bc	23.33 ± 1.43c	28.06 ± 1.44ab
TN (g/kg)	2.94 ± 0.15a	2.87 ± 0.11a	2.86 ± 0.02a	2.84 ± 0.14a	2.43 ± 0.14b	2.27 ± 0.18b
AP (mg/kg)	44.78 ± 6.77c	64.35 ± 7.58ab	47.88 ± 6.83bc	37.15 ± 3.12c	65.3 ± 4.60a	51.7 ± 6.46abc
AK (mg/kg)	249.33 ± 4.16d	296.67 ± 1.53c	161.67 ± 1.53e	237.33 ± 12.50d	708 ± 5.20a	500.33 ± 14.98b
EOC (mg/g)	5.89 ± 2.87a	2.32 ± 0.42b	3.23 ± 0.53ab	3.37 ± 0.85ab	3.84 ± 0.76ab	3.82 ± 0.44ab

The data in the table were mean ± standard deviation (*n* = 4), the different small letters indicated significant (*P* < 0.05) differences by one-way ANOVAs (Tukey’s multiple comparison test)

*EC* electrical conductivity, *SOM* soil organic matter, *TN* total nitrogen, *AP* available phosphorus, *AK* available potassium, *EOC* KMnO_4_-oxidizable carbon, *N* new ginseng field, *O* old ginseng field, *NB* new ginseng field without planting, *ND* new ginseng field with symptomatic American ginseng, *NH* new ginseng field with asymptomatic American ginseng, *OB* old ginseng field without planting, *OD* old ginseng field with symptomatic American ginseng, *OH* old ginseng field with asymptomatic American ginseng

### Fungal diversity and the relationship between soil physicochemical properties and community structure

**Fig. 1:**
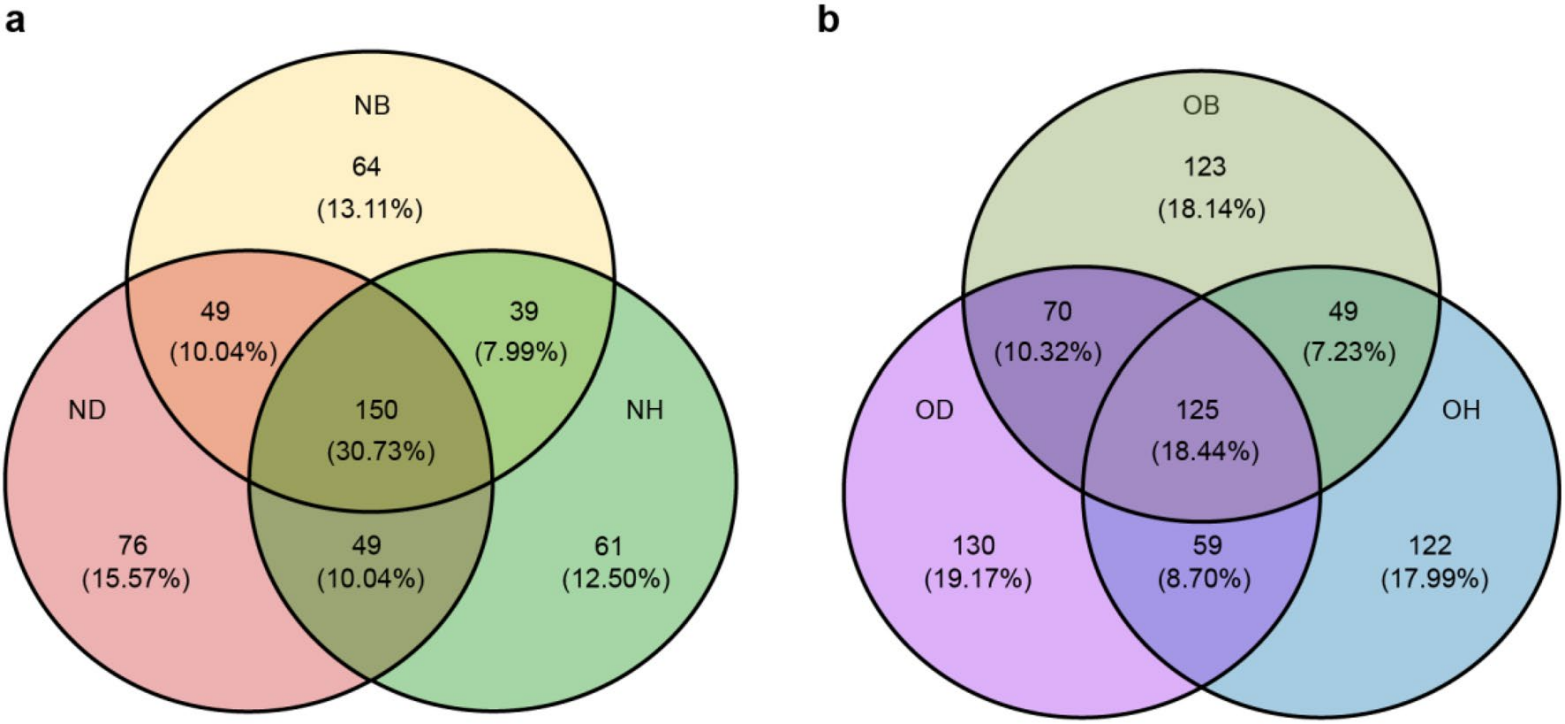
Differences between OTU distributions of new and old ginseng fields. *NB* new ginseng field without planting, *ND* new ginseng field with symptomatic American ginseng, *NH* new ginseng field with asymptomatic American ginseng, *OB* old ginseng field without planting, *OD* old ginseng field with symptomatic American ginseng, *OH* old ginseng field with asymptomatic American ginseng

**Fig. 2:**
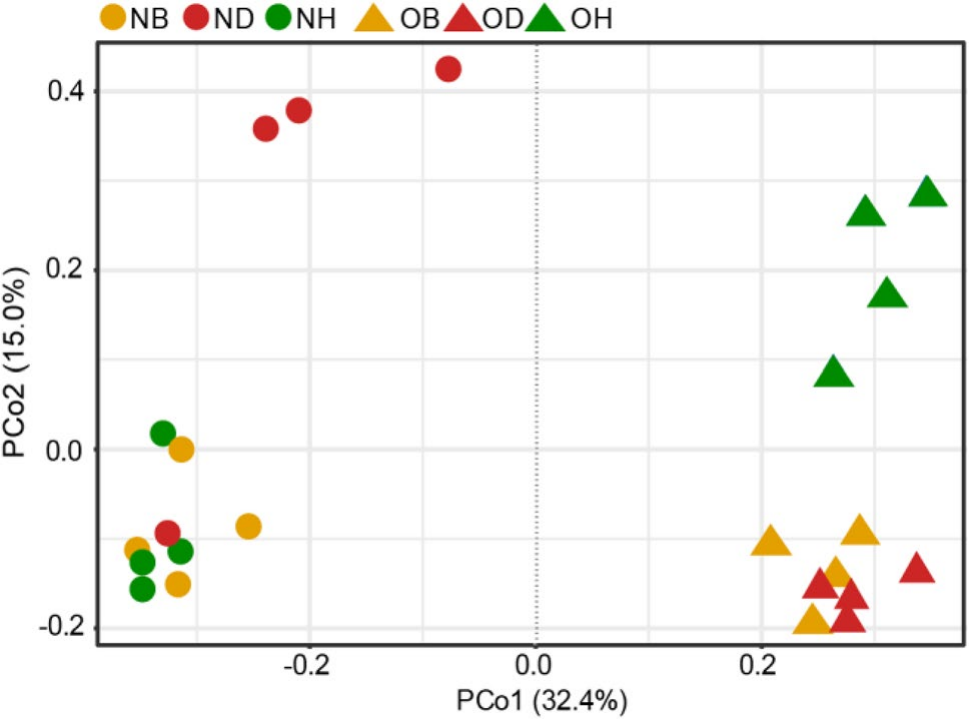
PCoA plot of fungal communities in new and old ginseng fields based on Bray–Curtis distance. *NB* new ginseng field without planting, *ND* new ginseng field with symptomatic American ginseng, *NH* new ginseng field with asymptomatic American ginseng, *OB* old ginseng field without planting, *OD* old ginseng field with symptomatic American ginseng, *OH* old ginseng field with asymptomatic American ginseng

Fungal α-diversity was determined based on the analysis of obtained fungal ITS sequences. By calculating Chao1 and ACE indices, no significant differences were observed concerning the richness of all samples. Additionally, Shannon and Simpson diversity indices recorded slight changes between new and old ginseng fields (Table S2). Moreover, Venn diagram results indicated that fungal OTUs detected in all three samples decreased from 30.73% in the new ginseng field to 18.44% in the old ginseng field (Fig. 1). In contrast, the numbers of unique fungi increased in the old ginseng field with respect to the new ginseng field (Fig. 1). Overall patterns of fungal communities in the new and old ginseng fields were further assessed using PCoA ordination based on Bray–Curtis distance. The first and second principal coordinates represented 32.4% and 15.0% of the total variance, respectively (Fig. 2), and a significant difference was observed between communities in both fields (PERMANOVA, *R*^2^ = 0.627, *P* < 0.001). The plot also showed that fungal communities in NH and NB samples were remarkably different from ND samples along the PCo2 axis. In comparison, OB and OD fungal communities were clustered (Fig. 2). This result may be related to the negative soil legacy in the old ginseng field. The relative abundances of potentially pathogenic fungi in all samples were examined using FUNGuild (Fig. S2). Results from this analysis showed that the relative abundances of potential plant pathogenic fungi in OB were significantly higher than those in OH, and no significant difference was observed compared with OD samples. These results indicate that the old ginseng field may have a negative soil legacy that affects the composition of fungal communities, and differences between fungal communities were affected by plant health status.

**Fig. 3:**
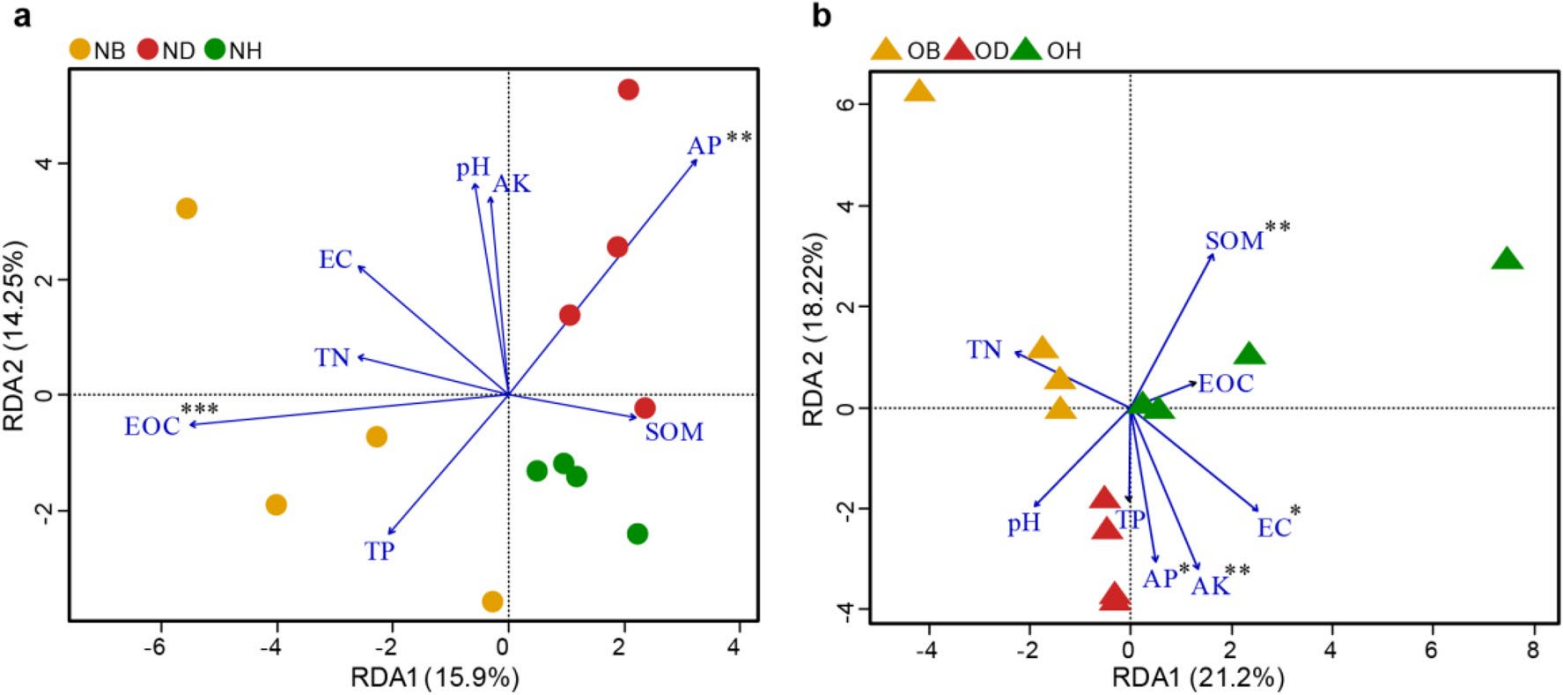
Redundancy analysis (RDA) showing the correlation between soil physicochemical properties and fungal communities based on OTUs. Arrow lengths are proportional to the influence of soil physical and chemical characteristics on the fungal community structure in **a** the new ginseng field, and **b** the old ginseng field. Asterisks indicate significant effects on the weighted data (*P < 0.05; **P < 0.01; ***P < 0.001). *EC* electrical conductivity, *SOM* soil organic matter, *TN* total nitrogen, *TP* total phosphorus, *AP* available phosphorus, *AK* available potassium, *EOC* KMnO_4_-oxidizable carbon, *NB* new ginseng field without planting, *ND* new ginseng field with symptomatic American ginseng, *NH* new ginseng field with asymptomatic American ginseng, *OB* old ginseng field without planting, *OD* old ginseng field with symptomatic American ginseng, *OH* old ginseng field with asymptomatic American ginseng

RDA was performed to investigate whether changes in community assemblages were affected by soil physicochemical properties (Fig. 3). Results indicate that the first two RDA axes could explain 30.15% and 39.42% of the total variances of the fungal community structure of the new and old ginseng field, respectively. For the community in the new ginseng field, NB samples were separated from NH and ND samples along the RDA1 axis (15.9%), and soil AP (*P* < 0.01) and EOC (*P* < 0.001) were found to be the major factors responsible for the shift of fungal communities (Fig. 3a). For the community in the old ginseng field, OB, OH, and OD were separated from each other, respectively (Fig. 3b). Additionally, fungal communities in the old ginseng field were significantly associated with soil characteristics, such as AK (*P* < 0.01), SOM (*P* < 0.01), soil AP (*P* < 0.05) and soil EC (*P* < 0.05; Fig. 3b). RDA results suggest that differences in fungal communities in the new and old ginseng fields were associated with different soil physicochemical factors and the composition of fungal communities in the old ginseng field was associated with more soil physicochemical factors (Fig. 3).

### Fungal abundance and composition

**Fig. 4:**
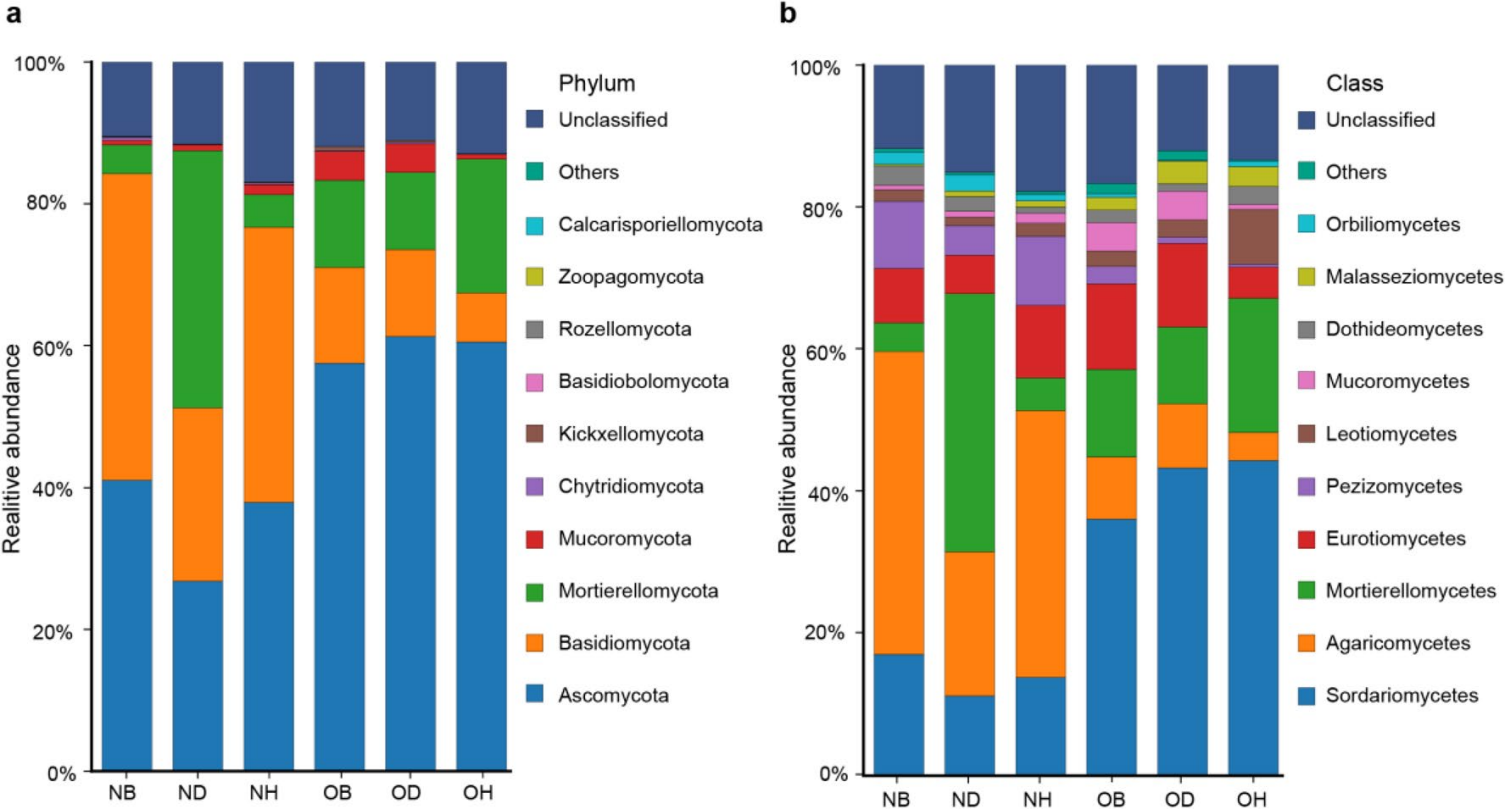
Relative abundances of **a** dominant fungal phyla, and **b** classes in the different samples. Only the top 10 predominant groups were plotted. *NB* new ginseng field without planting, *ND* new ginseng field with symptomatic American ginseng, *NH* new ginseng field with asymptomatic American ginseng, *OB* old ginseng field without planting, *OD* old ginseng field with symptomatic American ginseng, *OH* old ginseng field with asymptomatic American ginseng

To distinguish soil fungal taxa responses among the new and old ginseng fields, changes in the relative abundances of the primary (top 10) phyla and (top 10) classes were examined (Fig. 4). Variance analysis indicated that the relative abundance of Ascomycota was significantly higher in the old ginseng field (OH and OD samples) than in the new ginseng field (NH and ND samples) (Fig. 4a). The relative abundance of Basidiomycota was significantly lower in OH samples than in NH samples, and the relative abundance of Mortierellomycota was significantly lower in OD samples than in ND samples (Fig. 4a). High levels of fungi belonging to Sordariomycetes, Agaricomycetes, and Mortierellomycetes were identified via further taxonomical classification at the class level (Fig. 4b). The relative abundance of Sordariomycetes in the old ginseng field (OH and OD samples) were significantly higher than those in the new ginseng field (NH and ND samples), and the relative abundance of Agaricomycetes was significantly lower in OH samples compared to NH samples. The relative abundance of Mortierellomycetes was significantly lower in OD samples than in ND samples.

**Fig. 5:**
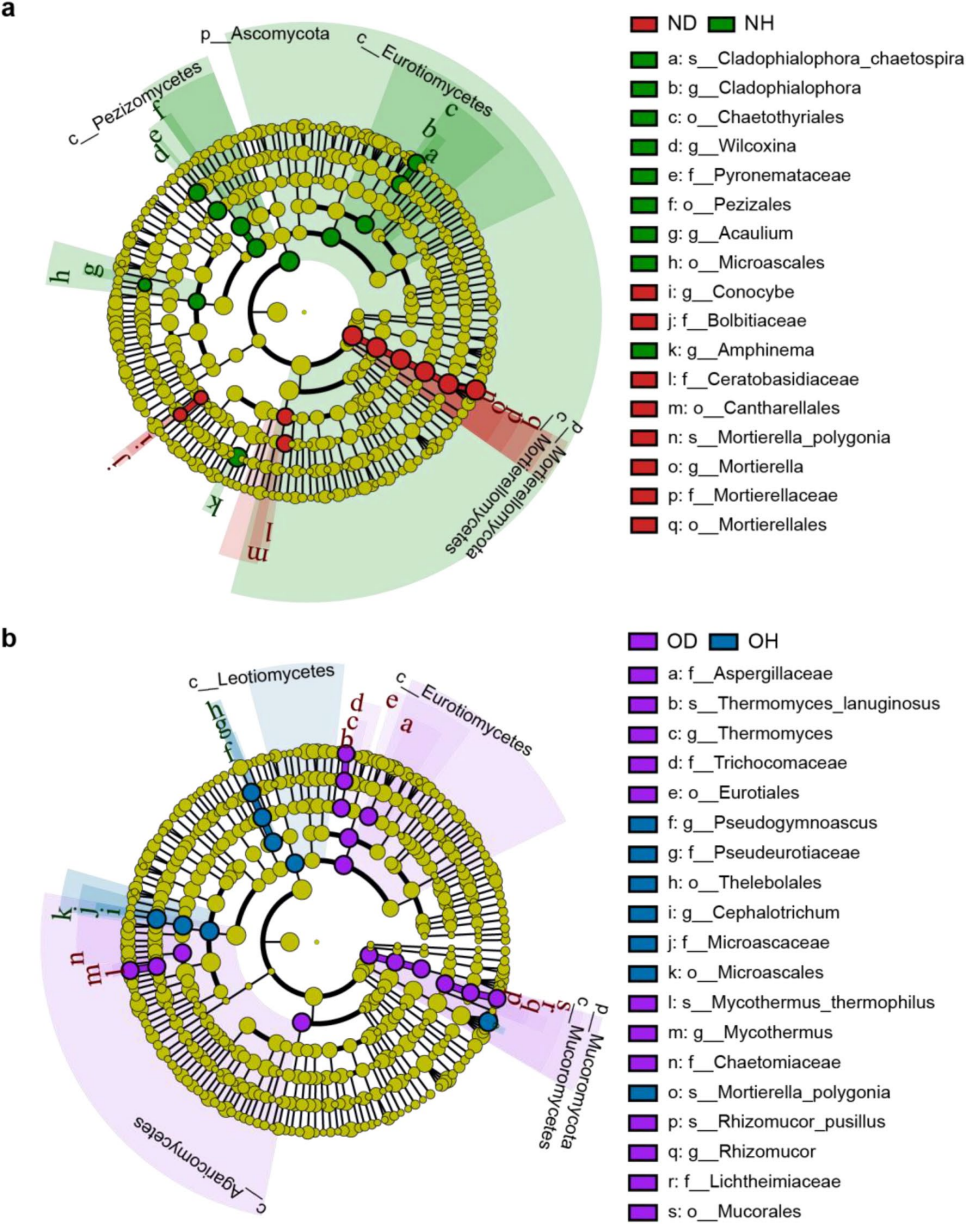
LEfSe analysis of fungal abundance for the different soil samples. Cladogram of fungal communities in **a** the new ginseng field, and **b** the old ginseng field. Only taxa having an LDA significance threshold > 4 are shown. Taxa with significantly different abundances among treatments are represented by colored dots. From the center outward, they represent the kingdom, phylum, class, order, family, genus, and species levels. Colored shadows represent trends of significantly different taxa. *ND* new ginseng field with symptomatic American ginseng, *NH* new ginseng field with asymptomatic American ginseng, *OD* old ginseng field with symptomatic American ginseng, *OH* old ginseng field with asymptomatic American ginseng (color figure online)

Differences in fungal composition between healthy and diseased groups in new and old ginseng fields at multiple taxonomic levels were examined. In the new ginseng field, LEfSe analysis indicated that fungal lineage was enriched in ND samples with Mortierellomycota (phylum) to *Mortierella_polygonia* (species), *Ceratobasidiaceae* (family) within Cantharellales (order), and from *Bolbitiaceae* (family) to *Conocybe* (genus) (Fig. 5a). Relative rich taxonomic groups in NH samples mainly contained the lineage from Chaetothyriales (order) to *Cladophialophora_chaetospira* (species), Pezizales (order) to *Wilcoxina* (genus) and Microascales (order), *Amphinema* (genus), and *Acaulium* (genus). Representative taxa in OD samples (Fig. 5b) included lineage from Mucoromycota (phylum) to *Rhizomucor_pusillus* (species), lineage from Eurotiales (order) to *Thermomyces_lanuginosus* (species) and *Aspergillaceae* (family) within Eurotiomycetes (class), as well as *Chaetomiaceae* (family) and *Mycothermus* (genus). Representative taxa in OH samples included lineage from Leotiomycetes (class) to *Pseudogymnoascus* (genus) and Microascales (order) to *Cephalotrichum* (genus). These results indicate that different pathogenic taxa in the new and old ginseng fields were tailored to specific microbial lineages.

### Fungal co-occurrence networks

**Fig. 6:**
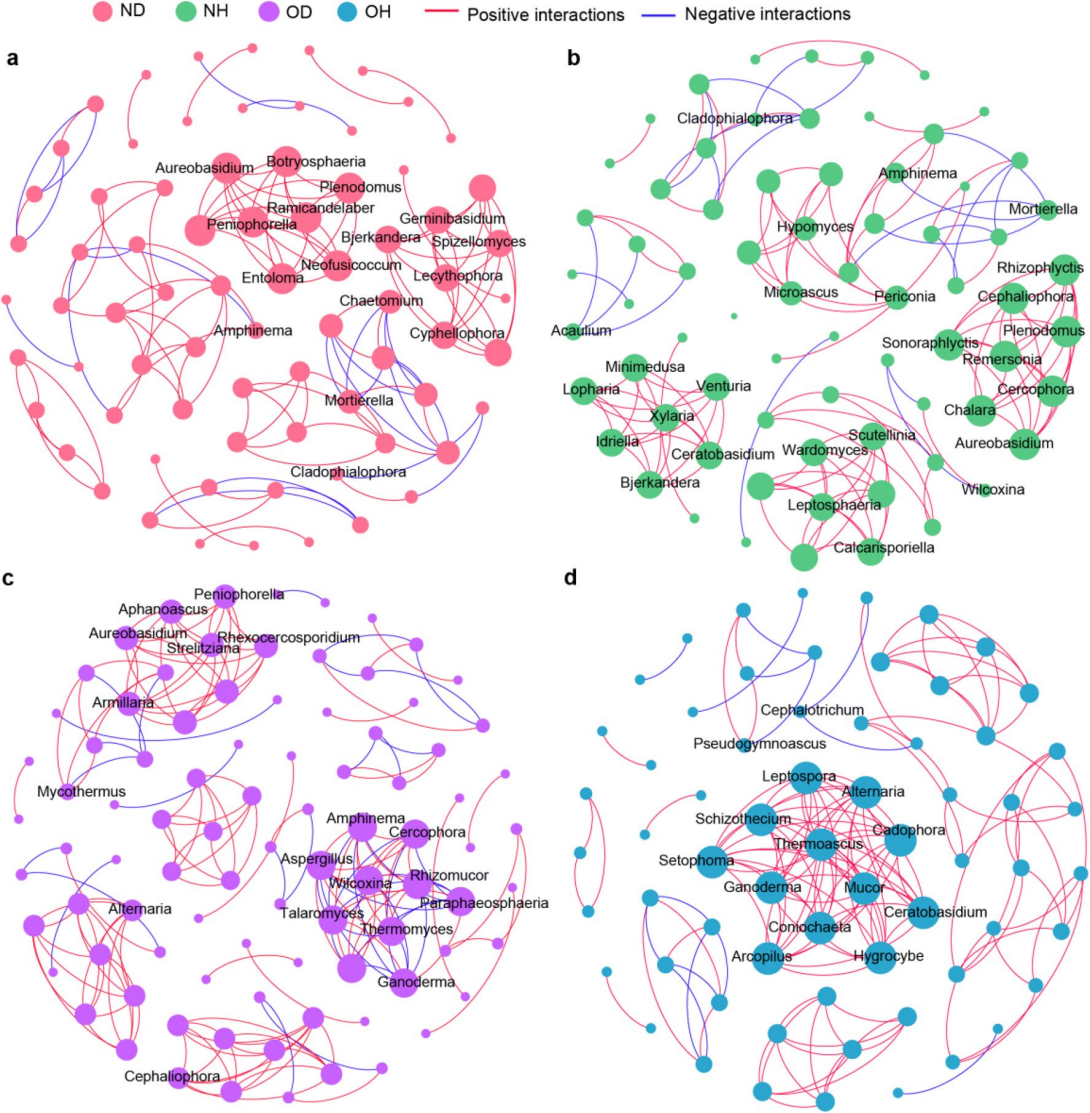
Co-occurrence networks of healthy and diseased American ginseng in new and old ginseng fields. Networks were constructed using pairwise Spearman’s rank correlation coefficients at the genus level (Spearman’s rho > 0.9, *P* < 0.05). Node size is proportional to degree. Labels only displayed parts of nodes with LefSe biomarkers or degrees ≥ 5. Positive (red edges) and negative (blue edges) correlations between nodes are shown. **a**–**d** Represent ND, NH, OD and OH samples, respectively. *ND* new ginseng field with symptomatic American ginseng, *NH* new ginseng field with asymptomatic American ginseng, *OD* old ginseng field with symptomatic American ginseng, *OH* old ginseng field with asymptomatic American ginseng (color figure online)

Fungal networks based on genus level were constructed to identify co-occurrence patterns of soil fungal community and niche partition in healthy and diseased groups in both new and old ginseng fields (Fig. 6). The size of each node was proportional to degree, and nodes with the highest degree were considered as keystone taxa. Node size was taken to reflect the relative abundance of the groups represented by the nodes. Results indicated that more positive correlations existed between nodes (indicated by red edges in Fig. 6) in all samples, and the ratio of negative correlations (blue edges) to positive correlations increased in diseased American ginseng, especially in old ginseng fields (OH N/P: 10.61%; OD N/P: 31.11%; Table S3). Based on multiple topological properties, fungal co-occurrence patterns in new and old ginseng fields after plants were infected resulted in a noticeable increase in the number of nodes; average degree and graph density decreased (Table S3). Here, after infection of American ginseng, especially in the old ginseng field, community interactions of competition intensified and density decreased.

It was also observed that keystones recorded noticeable differences in different samples. For example, *Lecythophora*, *Spizellomyces*, *Bjerkandera,* and *Cyphellophora*, amongst others, were keystones in ND (Fig. 6a), and *Aureobasidium*, *Cercophora,* and *Rhizophlyctis*, amongst others, were keystones in NH (Fig. 6b). *Armillaria*, *Aphanoascus*, *Aspergillus*, *Aureobasidium*, *Paraphaeosphaeria*, *Rhexocercosporidium,* and *Rhizomucor* were the keystones in OD (Fig. 6c), and *Alternaria*, *Setophoma*, *Cadophora,* and *Coniochaeta*, amongst others, were the keystones in OH (Fig. 6d).

### Prediction of fungal community ecological guilds

**Fig. 7:**
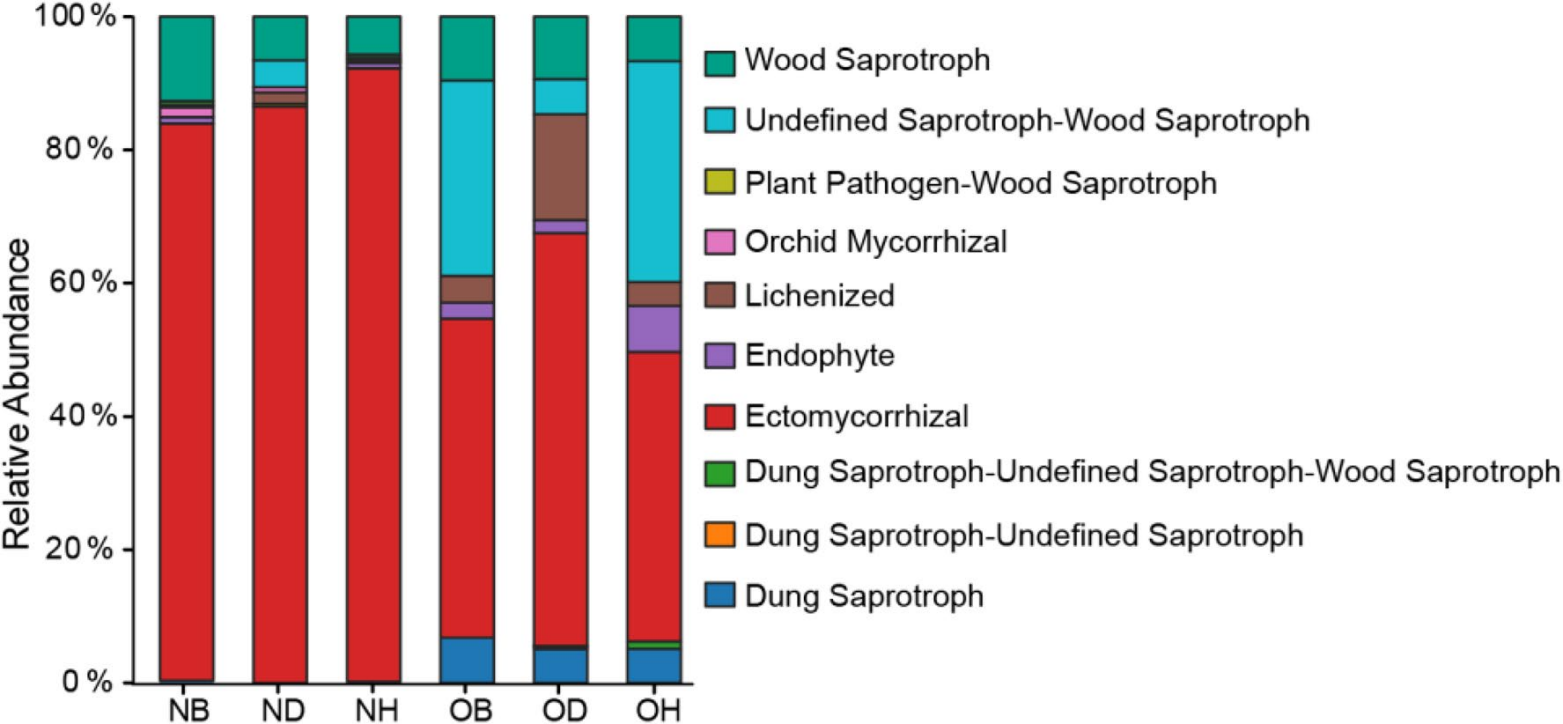
Comparison of the ecological guild of fungal communities according to different samples using FUNGuild. *NB* new ginseng field without planting, *ND* new ginseng field with symptomatic American ginseng, *NH* new ginseng field with asymptomatic American ginseng, *OB* old ginseng field without planting, *OD* old ginseng field with symptomatic American ginseng, *OH* old ginseng field with asymptomatic American ginseng

Ecological guilds of fungal taxa were predicted and functional changes of fungal communities in new and old ginseng fields were identified using FUNGuild (Fig. 7). Overall, 48.28% and 40.41% of OTUs from the new and old ginseng fields were assigned to different functional guilds, respectively. The relative abundance of top 10 trophic modes was analyzed in this study, among which ectomycorrhizal was the dominant functional guild in both new and old ginseng fields (Fig. 7). Additionally, the relative abundance of ectomycorrhizal was significantly lower in the old field compared to the new ginseng field (*P* < 0.05, Student’s *t* test). In contrast, the relative abundance of dung saprotroph, lichenized, and undefined saprotroph-wood saprotroph in the old ginseng field were significantly rich compared to the new ginseng field (*P* < 0.05, Student’s *t* test). Consistent with this finding, the relative abundance of lichenized in OD samples was significantly higher than that in OH samples in the old ginseng field (*P* < 0.05, one-way ANOVA-Tukey’s multiple comparison test). These results further confirm that fungal community ecological functions of American ginseng in different pathologies were different in the new and old ginseng fields.

## Discussion

### Different soil properties drive the composition/structure of the fungal community in new and old ginseng fields

Problems pertaining to replanting of ginseng in old ginseng fields have always been an area of great concern (Jiao et al. 2019; Li 1995). Knowledge of the diversity and structure of the fungal community during continuous cropping is essential to promote sustainable cultivation of ginseng (Tan et al. 2017). PCoA results indicated that fungal communities in OB and OD were clustered, while NB samples were gathered with NH samples in new ginseng field (Fig. 2). This indicates that the old ginseng field might have negative plant–soil legacy effects/feedbacks (Huang et al. 2013). According to previous investigations, these interactions between plants and their biotic and abiotic soil environment are called plant–soil feedbacks (Ehrenfeld et al. 2005). Moreover, plants in their own conspecific soil or other plants in the old field can induce soil legacy effects through changes in the composition of the associated soil microbial community (Bever 1994; van der Putten et al. 2013). These effects result in soil samples in the old ginseng field to have more unique fungi than those in the new ginseng field (Fig. 1), possibly related to soil legacy effects.

Another interesting finding is that fungal communities in old ginseng field samples were highly associated with soil physicochemical properties, indicating that fungal communities of the old ginseng field were more sensitive to soil environmental changes (Fig. 3). Specifically, soil AP and SOM were significantly associated with fungal community composition shifts in the old ginseng field (Fig. 3b). This finding is consistent with the findings of Lei et al. (2020), who highlighted that soil nutrients such as AP and SOM were the main drivers shaping soil fungal community composition in continuous monocropping of *Sophora flavescens*. It has also been recorded that phosphorus plays a pivotal role in ginseng root development and increasing disease resistance (Konsler and Shelton 1990; Li 1995). Fang et al. (2012) found that SOM inputs can significantly reduce the severity of strawberry (*Fragaria* × *ananassa*) *Fusarium* wilt caused by plant pathogens in natural systems. In our results, SOM content in OD samples was significantly lower than those in OH samples (Table 1), indicating that plant diseases in the old ginseng field might be related to low SOM. Additionally, soil AK was also found to be a dominant factor shifting the soil fungal communities in the old ginseng field (Fig. 3b). Moreover, soil AK in the old ginseng field was significantly higher than that in the new ginseng field (Table 1), consistent with the findings by Fu et al. (2009). Although the amount of potassium fertilizer applied to the new and old ginseng fields was the same, the amount of potassium absorbed by American ginseng grown in the old ginseng field might be less than the applied concentration.

Soil pH and TN surrounding ginseng samples in the old ginseng field were significantly lower than those in the new ginseng field (Table 1). Qi et al. (2019) showed that soil pH is inversely proportional to soil-borne diseases, and lower pH levels and high levels of phenolic acid have been reported under continuous ginseng cropping (Li et al. 2015; You et al. 2015). In addition, Du et al. (2011) recorded phenolic acids to be negatively correlated with soil nitrogen. It is therefore reasonable to assume that lower soil TN levels could be recorded in old ginseng field than in new ginseng field. This assumption was confirmed by findings from our study (Table 1), as well as in the findings of Dou et al. (2018) with a recorded decline in soil TN with cropping years. In conclusion, to maximize the use of old ginseng fields for continuous cropping, TN and SOM content in old fields need to be increased; improving ginseng plant nutrient utilization is therefore one strategy to control replanting diseases.

### Specific fungal taxonomic groups in new and old ginseng fields have led to differences in community structure

Based on our results, it is evident that fungal taxonomic composition strongly varied between the new and old ginseng fields (Fig. 4). Compared to results from the new ginseng field, Ascomycota was the most dominant phylum in the old ginseng field, especially in OD samples (Fig. 4a). The previous study by Berbee (2001) highlighted that many destructive fungal pathogens belong to the Ascomycetes phylum. However, in Mortierellomycota and Basidiomycota, some members of genera *Mortierella*, *Amphinema*, *Tomentella*, and *Lactarius* have been reported to be beneficial to plant growth (Cairney and Chambers 1999; Jones et al. 2009; Xiong et al. 2017), and their relative abundances were reduced compared to new ginseng field. Supporting this observation, it has been previously reported that the relative abundance of Basidiomycota and Mortierellomycota were higher in healthy peanut (*Arachis hypogaea* L.) genotype plants with respect to diseased genotypes (Kusstatscher et al. 2019; Li et al. 2019). These findings indicate that the susceptibility of American ginseng in the old ginseng field to disease is likely to be associated with changes in fungal community composition.

Fungal biomarkers in the new and old ginseng fields were examined using LEfSe analysis. Previous studies reported ectomycorrhizal fungi of *Wilcoxina* and *Amphinema* to assist host plants in nitrogen uptake (Jones et al. 2009), and *Cladophialophora* to be a well-known dark septate endophytic fungus that can benefit host plants by promoting phosphorus and nitrogen uptake (Usuki and Narisawa 2007). Enrichment of *Wilcoxina*, *Amphinema,* and *Cladophialophora* in this study indicated that, in the new ginseng field, healthy American ginseng recruited more fungi with nutrient cycling properties than diseased American ginseng. In contrast, *Conocybe* and *Mortierella* genera were significantly enriched in ND samples (Fig. 5a). Members of *Conocybe* have been reported to grow on a variety of substrates, typically on rotten wood, moss, and rotten grass, as well as dung, and occasionally attaching to plant remains and vegetable refuse (Amandeep 2013). Previous studies have shown that some species of *Mortierella* can produce antibiotics, and several strains have been investigated as potential antagonistic agents that can be used against various plant pathogens (Tagawa et al. 2010; Wang et al. 2018a, b). Findings by Hernández et al. (2018), however, showed that some members of *Mortierella* are pathogenic to avocado crops. The *Mortierella* spp*.* can be recruited by diseased plants as an antifungal agent, which may also act as an opportunistic pathogen in diseased ginseng plants. An increase of *Conocybe* and *Mortierella* could, therefore, be used as an indicator of diseased American ginseng in the new ginseng fields. One possible explanation for the increased abundance of specific fungi in ND samples is that plants participate in shaping the fungal community composition to protect themselves against invading pathogens (Berendsen et al. 2012; Huang et al. 2019).

The genera *Pseudogymnoascus* and *Cephalotrichum* were the main biomarkers recorded in OH samples (Fig. 5b)*.* Tajuddin et al. (2017) highlighted that *Pseudogymnoascus* can use organic forms of nitrogen, as well as degrade cellulose. Deng et al. (2018) recorded some species of *Cephalotrichum* to be useful in controlling root-rot disease and assisting in promoting *P. notoginseng* or *P. ginseng* growth. In contrast, *Mycothermus*, *Rhizomucor*, and *Thermomyces* genera increased in OD samples (Fig. 5b). It is currently known that *Mycothermus* acts as a key factor in organism decay of plant materials and plant-derived commodities (Cannon and Kirk 2007). Additionally, a thermophilic fungus in the *Chaetomiaceae* family is a well-known producer of cellulases and hemicellulases (Basotra et al. 2016). Although direct evidence regarding the effect *Mycothermus* has on plant disease is lacking, García-Estrada et al. (2005) implied that members of *Chaetomiaceae* are potential antagonists toward many soil-borne pathogens. Additionally, *Rhizomucor* may have an indirect effect on American ginseng by producing phytase to promote its uptake of phytate phosphorus (Singh and Satyanarayana 2011). Moreover, several strains of the *Thermomyces* genus have been found to be hyper producers of extracellular xylanase (Singh et al. 2003). In general, similar to results for the new ginseng field, OH soil recruited more fungal groups involved in nutrient cycling, likely to promote plant growth, while OD samples, decomposing enzymes, and antagonistic fungi significantly increased, possibly being related to metabolism and root exudates in American ginseng.

### Interactions and functional differences of the fungal community in new and old ginseng fields

In an ecosystem, different populations undertake different types of interactions, such as competition and mutualism, forming complicated networks (Deng et al. 2012). In our study, after American ginseng had become infected, especially in the old ginseng field, the proportion of negative to positive correlations increased; the number of nodes also recorded a significant increase, and average degree and graph density decreased (Table S3). These results indicate that competition of fungi was stronger and synergism was weaker in soil in the old ginseng field, especially after infection. Additionally, keystone species frequently co-occurred with other groups, possibly exerting a strong influence on the structure of fungal communities (Agler et al. 2016; Hassani et al. 2018). For example, during continuous ginseng cropping in the old ginseng field, *Alternaria*, *Setophoma* and *Cadophora* genera were the main keystones in OH soil (Fig. 6c). Although *Alternaria* genus has been previously documented as major plant pathogens, they also cause leaf and stem blight of American ginseng (Quayyum et al. 2005). Additionally, *Setophoma* species have been reported to cause root-rot, wilting and other infections in many different vegetables (Ikeda et al. 2012; Yang et al. 2017). Khastini et al. (2014) recorded *Fusarium* wilt in *Cucumis melo* L. to be controlled by *Cadophora*, suggesting that some fungal pathogens in the old ginseng field could have multi-connectivity with other groups, although the ginseng plants were healthy. Fortunately, some microbial groups, for example *Cadophora*, are likely to play a positive role in maintaining plant health in the old ginseng field. *Armillaria, Aphanoascus, Aspergillus,* and *Rhexocercosporidium* are soil pathogenic fungi that can cause many plant diseases (Bai et al. 2018; Baumgartner 2004), with *Rhexocercosporidium* being a devastating causal agent causing rusted root of American ginseng (Reeleder et al. 2006). These pathogenic genera were the keystones in the OD soil (Fig. 6d). Collectively, these results indicate that an imbalance in the soil fungal community relationship, as well as active fungal pathogens, may result in diseased American ginseng in the old ginseng field.

Based on these findings, it is evident that fungal diversity, composition, and community interactions as soil legacy effects can affect replanting in old ginseng fields. Additionally, functional guild constitutes a further biological feature of fungi that could predict their activity in ecosystems (Talbot et al. 2015). Functional guild results in our study for dung saprotroph, lichenized, and undefined saprotroph-wood saprotroph which were higher in the old ginseng field than in the new field; the functional guild of ectomycorrhizal fungi in the old ginseng field was lower than that in the new ginseng field (Fig. 7). The majority of increases in functional guild included saprotrophs, with previous studies highlighting that long-term continuous cultivation could enrich saprotrophs (Li et al. 2020b; Liu et al. 2019). However, ectomycorrhizal plays an active role in the transformation of soil organic matter, nitrogen, and phosphorus, which were relatively less abundant in the old ginseng field (Landeweert et al. 2001; Lindahl and Tunlid 2015). Therefore, changes of fungal trophic strategies, i.e., fungal-mediated ecological functions (such as decomposition and nutrient cycling), is a possible reason for difficulties in replanting ginseng in old ginseng fields.

## Conclusions

In conclusion, our study presented soil physicochemical properties and soil fungal community dynamics in relation to replant failure, determining the response of soil legacy effects in old ginseng field to replanting diseases. We suggest that the management of replanting American ginseng needs to focus on proper fertilization, such as increasing the soil nutrition of TN and SOM, as well as reasonably regulating soil pH. While paying attention to fungal communities' response to replanting diseases, changes in bacterial communities should also be considered. The isolation and identification of potential bio-control agents/biological soil amendments (such as *Amphinema, Cladophialophora*, *Cadophora*, *Mortierella*, and *Wilcoxina*) from suppressive soil and whether they could be used as bio-fertilizers to stabilize the interactions between microbial communities and improve disease resistance also needs further investigation.

## Supplementary Information

Below is the link to the electronic supplementary material.

Supplementary file1 (DOC 128 KB)
