# Supplementary material for: Impacts of replanting American ginseng on fungal assembly and abundance in response to disease outbreaks
Source: Arch Microbiol. 2021 Feb 22;203(5):2157–70. doi: 10.1007/s00203-021-02196-8 (PMC8205870; doi:10.1007/s00203-021-02196-8)
Supplement: Supplementary file 1 — Supplementary file1 (DOC 128 KB) [file 203_2021_2196_MOESM1_ESM.doc]

**Table S1.** The quality assessment statistics of sequencing data.

| Sample | PE Reads | Raw Tags | Clean Tags | Effective Tags | AvgLen | GC (%) | Q20 | Q30 | Effective |
| --- | --- | --- | --- | --- | --- | --- | --- | --- | --- |
|  |  |  |  |  | (bp) |  | (%) | (%) | (%) |
| NB | 466083 | 119726 | 119726 | 119322 | 258 | 50.64 | 99.64 | 98.33 | 25.6 |
| ND | 494963 | 96875 | 96875 | 96616 | 247 | 44.93 | 99.72 | 98.61 | 19.52 |
| NH | 541118 | 101189 | 101189 | 100909 | 256 | 49.93 | 99.52 | 98.16 | 18.65 |
| OB | 424892 | 76578 | 76578 | 76410 | 252 | 50.46 | 99.67 | 98.46 | 17.98 |
| OD | 471218 | 103089 | 103089 | 102580 | 249 | 51.4 | 99.74 | 98.68 | 21.77 |
| OH | 428115 | 127536 | 127536 | 127313 | 251 | 48.92 | 99.68 | 98.54 | 29.74 |

PE Reads: the number of paired-end reads; Raw Tags: the number of original sequences obtained by paired-end reads; Clean Tags: the number of optimized tags filtered for the raw tags; Effective Tags: the number of effective tags after filtering the chimera by clean Tags; AvgLen (bp): the average sequence length; GC (%): the percentage of G and C type bases to total bases; Q20 (%): the percentage of bases with quality greater than or equal to 20 in the total base number; Q30 (%) is the percentage of bases with quality greater than or equal to 30 in the total base number; Effective(%): Effective Tags as a percentage of PE Reads.

NB: new ginseng field without planting; ND: new ginseng field with symptomatic American ginseng; NH: new ginseng field with asymptomatic American ginseng; OB: old ginseng field without planting; OD: old ginseng field with symptomatic American ginseng; OH: old ginseng field with asymptomatic American ginseng.

**Table S2.** The number of species at different taxonomic levels and α-diversity indices of different samples.

|  | N | | |  | O | | |
| --- | --- | --- | --- | --- | --- | --- | --- |
| Sample | NB | ND | NH |  | OB | OD | OH |
| Phylum | 7 | 6 | 8 |  | 6 | 6 | 5 |
| Class | 14 | 13 | 15 |  | 17 | 16 | 13 |
| Order | 30 | 31 | 33 |  | 35 | 38 | 32 |
| Family | 58 | 56 | 55 |  | 62 | 70 | 52 |
| Genus | 67 | 71 | 68 |  | 75 | 87 | 63 |
| Species | 47 | 52 | 51 |  | 59 | 63 | 48 |
| OTU_S_ | 248 | 238 | 248 |  | 305 | 303 | 281 |
| Chao1 | 164.20±14.72a | 176.63±27.57a | 171.27±10.03a |  | 200.56±23.61a | 183.28±18.66a | 205.37±45.04a |
| ACE | 159.83±14.95a | 174.78±25.30a | 159.16±8.20a |  | 196.93±17.38a | 180.72±16.25a | 203.98±62.74a |
| Shannon | 3.22±0.54b | 2.89±0.40b | 3.46±0.13ab |  | 4.17±0.16a | 3.53±0.56ab | 3.36±0.46ab |
| Simpson | 0.14±0.11ab | 0.20±0.11a | 0.09±0.03ab |  | 0.03±0.01b | 0.11±0.11ab | 0.09±0.04ab |

The data of Chao1, ACE, Shannon and Simpson indices data in the table were mean ± standard deviation (n = 4), and different small letters indicated significant (*P* < 0.05) differences by one-way ANOVAs (Tukey’s multiple comparison test).

N: new ginseng field; O: old ginseng field. NB: new ginseng field without planting; ND: new ginseng field with symptomatic American ginseng; NH: new ginseng field with asymptomatic American ginseng; OB: old ginseng field without planting; OD: old ginseng field with symptomatic American ginseng; OH: old ginseng field with asymptomatic American ginseng.

**Table S3.** The multiple topological properties of co-occurrence networks in different samples.

|  | ND | NH |  | OD | OH |
| --- | --- | --- | --- | --- | --- |
| Average degree | 3.43 | 3.87 |  | 3.98 | 4.56 |
| density | 0.047 | 0.056 |  | 0.045 | 0.072 |
| modularity | 0.885 | 0.88 |  | 0.868 | 0.759 |
| nodes | 74 | 70 |  | 89 | 64 |
| Edges (Negative/Positive) % | 23.3 | 20.35 |  | 31.11 | 10.61 |

ND: new ginseng field with symptomatic American ginseng; NH: new ginseng field with asymptomatic American ginseng; OD: old ginseng field with symptomatic American ginseng; OH: old ginseng field with asymptomatic American ginseng.

**
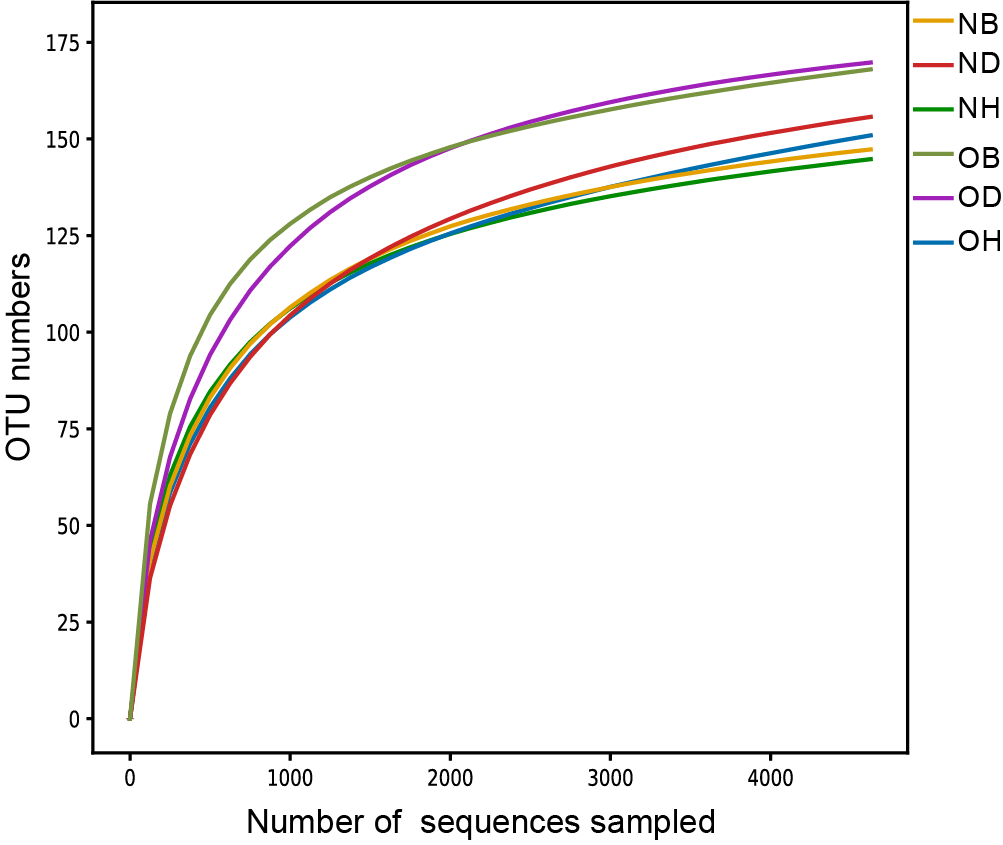
**

**Figure S1.** Rarefaction curves of OTU numbers against the total number of sequence reads.

NB: new ginseng field without planting; ND: new ginseng field with symptomatic American ginseng; NH: new ginseng field with asymptomatic American ginseng; OB: old ginseng field without planting; OD: old ginseng field with symptomatic American ginseng; OH: old ginseng field with asymptomatic American ginseng.


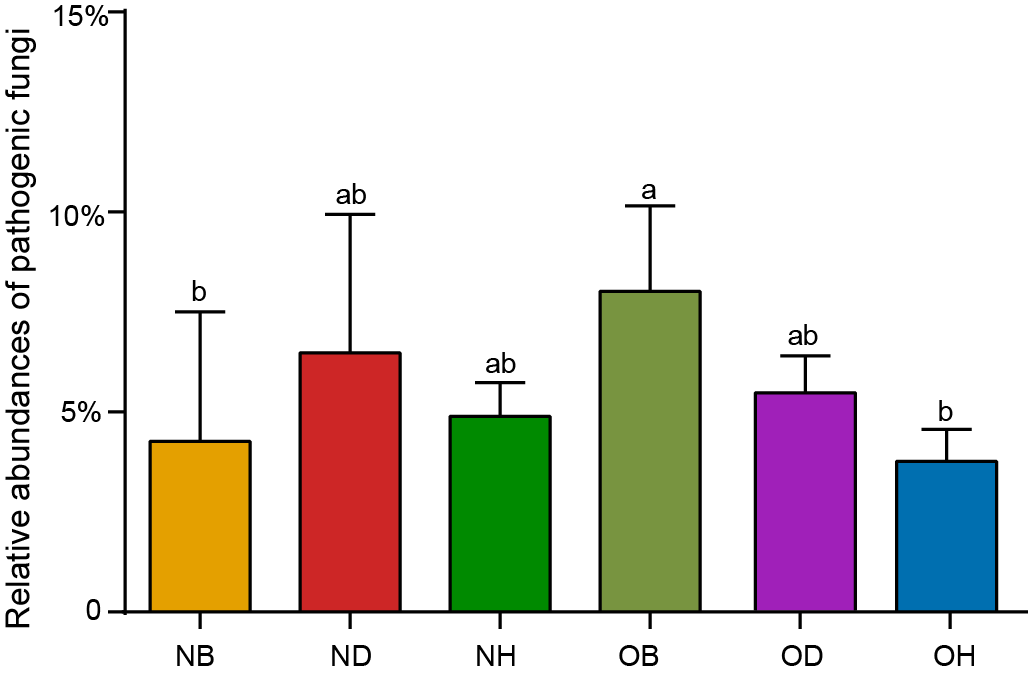


**Figure S2.** The comparison of the relative abundances of potentially pathogenic fungi in different soil samples.

NB: new ginseng field without planting; ND: new ginseng field with symptomatic American ginseng; NH: new ginseng field with asymptomatic American ginseng; OB: old ginseng field without planting; OD: old ginseng field with symptomatic American ginseng; OH: old ginseng field with asymptomatic American ginseng.
